# Effectiveness of Hyperbaric Oxygen for Fibromyalgia: A Meta-Analysis of Randomized Controlled Trials

**DOI:** 10.3390/clinpract13030053

**Published:** 2023-04-26

**Authors:** Chunfeng Cao, Qianlu Li, Xinran Zhang, Giustino Varrassi, Haiqiang Wang

**Affiliations:** 1 Department of Orthopedics, The Yongchuan Hospital of Chongqing Medical University, 439# Xuanhua Road, Yongchuan, Chongqing 402160, China; 2 Department of Neurology, The Yongchuan Hospital of Chongqing Medical University, 439# Xuanhua Road, Yongchuan, Chongqing 402160, China; 3 Department of Research, Polo Procacci Foundation, Via Tacito 7, 00193 Roma, Italy; 4 Institute of Integrative Medicine, Shaanxi University of Chinese Medicine, Xixian Avenue, Xixian District, Xi’an 712046, China

**Keywords:** hyperbaric oxygen therapy, fibromyalgia, systematic review, meta-analysis

## Abstract

Background: Hyperbaric oxygen therapy (HBOT) has been reported as an emerging treatment regimen for fibromyalgia syndrome (FMS), with a paucity of solid evidence. Accordingly, a systematic review and meta-analysis were performed to address the effectiveness of HBOT on FMS. Methods: We searched the Cochrane Database, EMBASE, Medline, PubMed, Clinicaltrials.gov, and PsycINFO, and the reference sections of original studies and systematic reviews from inception to May 2022. Randomized controlled trials (RCTs) on the treatment of FMS with HBOT were included. Outcome measures included pain, Fibromyalgia Impact Questionnaire (FIQ), Tender Points Count (TPC), and side effects. Results: Four RCTs, with 163 participants, were included for analysis. Pooled results showed that HBOT could benefit FMS with significant improvement at the end of treatment, including FIQ (SMD = −1.57, 95% CI −2.34 to −0.80) and TPC (SMD = −2.50, 95% CI −3.96 to −1.05). However, there was no significant effect on pain (SMD = −1.68, 95% CI, −4.47 to 1.11). Meanwhile, HBOT significantly increased the incidence of side effects (RR = 24.97, 95% CI 3.75 to 166.47). Conclusions: Collectively, emerging evidence from RCTs indicates that HBOT can benefit FMS patients in FIQ and TPC throughout the observation time phrases. Although HBOT has some side effects, it does not cause serious adverse consequences.

## 1. Introduction

Fibromyalgia syndrome (FMS) is characterized by chronic widespread skeletal muscle pain. It is the third most common musculoskeletal disorder after lumbar pain and osteoarthritis [1,2]. FMS patients can present with a range of dysfunction, including persistent fatigue, sleep disorders, cognitive retardation, functional bowel disease, paresthesias, and mood disturbance [3,4]. The prevalence of FMS in the general population is 0.2–6.6% [5], predominantly in females and three times higher than that of males [6]. The treatment of FMS is varied with indefinite curative effects [7]. Previous studies [8] have shown that pharmacological interventions are moderately effective for FMS. In spite of multiple medication options, most patients still experience pain restricting movement. It has become increasingly recognized that non-pharmacological interventions should be considered as complementary therapy for chronic pain added to a multidisciplinary treatment approach [4,7,9,10], such as aerobic exercise, meditative exercise therapy, and cognitive behavioral therapy. 

A non-invasive treatment regimen as hyperbaric oxygen therapy (HBOT) provides 100% oxygen at a pressure greater than that at sea level. Peripheral tissue hyperoxia promotes various biochemical effects that are beneficial in conditions such as infections, ischemia, and wound healing. In the past 20 years, HBOT has been used to treat more than 100 diseases worldwide, despite scant scientific evidence of its benefits and safety [11]. Recently, HBOT has shown good efficacy in the treatment of cluster headaches and migraines, idiopathic trigeminal neuralgia, complex regional pain syndrome, FMS, and other aspects [12,13,14,15,16]. It may alter the glial function and correct abnormal brain activity associated with FMS through anti-inflammatory effects [17,18,19].

EULAR recommendations for the management of FMS were updated in 2017 based on systematic reviews [7]. Due to insufficient evidence on medical efficacy, HBOT is not recommended by the recommendations, although it has been successfully used in the treatment of fibromyalgia. As well, there have been no systematic reviews summarizing emerging studies on HBOT in FMS until now. Therefore, we aimed to verify the efficacy and safety of HBOT in FMS by conducting a meta-analysis.

## 2. Methods

Because the study is a systematic review, approval from an ethics committee is not required. No systematic review protocol was published prior to the conclusion of this study. International Prospective Register of Systematic Reviews (PROSPERO) was used to register this systematic review and meta-analysis and was successful on 9 May 2021 (Registration No. CRD42021247078)

### 2.1. Study Selection

We conducted a systematic electronic search in Cochrane Database, EMBASE, Medline, PubMed, Clinicaltrials.gov, and PsycINFO to identify studies published before 21 May 2022 using the following strategy: “(FMS OR fibromyalgia OR musculoskeletal disease) AND (HBOT OR hyperbaric oxygenation OR hyperbaric oxygen therapy)” without language restrictions. Search strategies were tailored for each database. Three independent authors (QLL, XRZ, and CCF) screened study titles and abstracts and read the full text to assess study eligibility. Eligible studies were limited to RCTs.

### 2.2. Eligibility Criteria

The following standards were required: (1) adult patients (>18 years of age) with FMS based on the American College of Rheumatology (ACR) criteria [20]; (2) the type of intervention as compared HBOT with no treatment or other treatment options; (3) outcome measures reporting at least one items related to FMS symptom after follow up, including pain, tender points count (TPC), press pain threshold, fibromyalgia impact questionnaire (FIQ) and side effects; (4) the study was published in full paper form, and type of studies limited to RCTs.

Clinical controlled studies, retrospective and cohort studies were excluded. First, the headings are filtered to exclude duplicate studies. Subsequently, a careful review of the abstract and full text with the elimination of irrelevant papers. 

### 2.3. Data Collection Process

Two authors (CFC and QLL) extracted the data independently and repeatedly using Microsoft Office Excel 2007, which was piloted between the reviewers for consistency and accuracy. After reviewing the content of each article, the reviewer independently extracts the information according to the entries shown in the table. In cases where the literature was insufficient for analysis, we emailed the authors for more information. When the data of two researchers are inconsistent, a third author (KLM) is invited to participate in data extraction and discussion.

### 2.4. Rik of Bias (RoB) in Individual Studies

Cochrane Collaboration’s ROB 2 assessment instrument assessed the risk of bias of the recruited articles by two authors (QLL and XRZ) [21]. Five domains of bias were assessed by Cochrane Collaboration’s RoB 2: detect randomization process, intended interventions, missing outcome data, the measurement of the outcome, and the selection of reported results. RoB 2 makes judgments about the direction of bias for each domain and the overall. The study will be judged to be at low, moderate, or high ROBs if all domains measures are at low ROBs, at least one domain measure is at moderate ROBs (no measures with high ROBs), and one measure at high ROBs or multiple measures with substantially lowers confidence, respectively.

### 2.5. Assessment of Heterogeneity

Prior to the meta-analysis, we used standard Cochran’s Q tests and the I^2^ statistic to assess heterogeneity between compare trials. A statistically significant threshold was required to a *p* value < 0.05. The interpretation of I^2^ values is based on the Deeks [22]. The higher the I^2^, the more heterogeneity will be. The value of I^2^ has four levels, which were 0–40% (not important), 30–60% (‘moderate’ heterogeneity), 50–90% (‘substantial’ heterogeneity), and 75–100% (‘considerable’ heterogeneity), respectively. 

### 2.6. Assessment of Treatment Effect

RevMan 5.3 software was used for all statistical analyses. When data were continuous, a random-effects model was used to calculate the standardized mean difference (SMD). All effect sizes were determined by 95% confidence intervals (CIs).

## 3. Results

### 3.1. Hallmarks of Included Studies

We preliminarily reviewed 1295 relevant studies (Table 1) by PubMed (1137 citations), EMBASE (66 citations), Cochrane (52 citations), Ovid Medline (19 citations), Ovid PsycINFO (5 citations), and Clinicaltrail.gov (16 citations). Title and abstract were used as preliminary screening methods, and 881 were excluded. Five studies eventually were selected, four of which were included in this meta-analysis. In the absence of a control group, one study was excluded [23]. 

### 3.2. Participant Hallmarks

**Figure 1:**
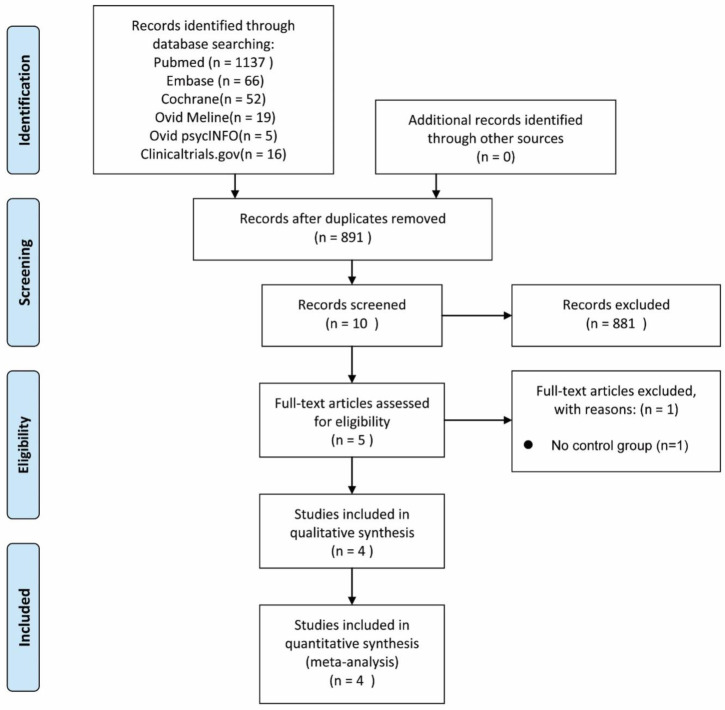
Flowchart of the literature screening process.

In total, 163 participants were included, including 82 patients undergoing HBOT intervention and 81 controls. In two of the studies, all the cases were women. The average observational time was 8.25 weeks (range, 3 weeks to 3 months). The reported age of participants was included in three studies involving the HBOT group and three studies involving the control group. The mean age for the HBOT group was 46.38, while the mean age for the control group was 43.69. The four studies included data from three countries. Two occurred in Israel, one in Turkey, and one in Spain. Table 2 shows a detailed list of study hallmarks. Figure 1 demonstrates the flowchart of the screening process.

### 3.3. ROBs of Included Studies (Figure 2)

None of the four studies had low ROBs. There are two studies at high risk of bias, as they had at least one high judgment in the key domains [19,24]. The remaining two studies were designated at some concerns (unclear intended interventions and randomization process) [12,25].

### 3.4. Effects of HBOT Interventions

So far, there has been no consensus regarding therapy sessions of HBOT. Amongst four included studies, two intervention sessions were 40 daily sessions, one was 60 daily sessions, and one was 15 sessions; all interventions were 5 days/week and 90 min each. A detailed list of HBOT (including pressure, therapy time, and therapy session) is shown in Table 2. 

### 3.5. Primary Outcome Measures

#### 3.5.1. Pain

**Figure 3:**
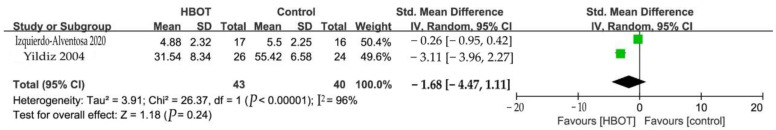
Forest plot for the comparison of pain at the end of treatment, showing the effect not favoring hyperbaric oxygen therapy [12,25].

Two studies used VAS (Visual Analogue Scale) scores to assess pain and showed the difference between HBOT and control groups at two weeks at the end of HBOT sessions (Figure 3). VAS had no statistical difference effect (SMD = −1.68, 95% CI, −4.47 to 1.11, *p* = 0.24). Considerable heterogeneity among these studies (I^2^ = 96%) was revealed by test statistics and adopted the random-effects model.

#### 3.5.2. Fibromyalgia Impact Questionnaire (FIQ)

**Figure 4:**
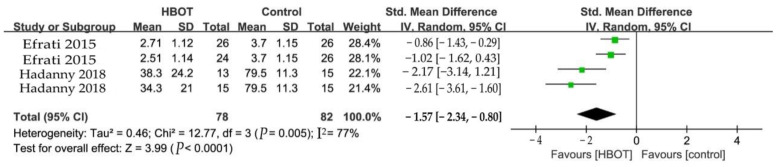
Forest plot for the comparison of FIQ at the end of treatment, showing the effect favoring hyperbaric oxygen therapy [19,24].

Functional capacity in daily-living activities was evaluated by the FIQ [26]. Two studies of the 4 RCTs used the FIQ as outcome measures at the end of HBOT sessions (Figure 4). And patients were randomly assigned to treat and crossover groups in two studies. HBOT improved FIQ by −157% when compared with controls (SMD = −1.57, 95% CI −2.34 to −0.80, *p* < 0.0001). Considerable heterogeneity was identified among these studies (I^2^ = 77%), and a random-effects model was adopted.

#### 3.5.3. Tender Points Count (TPC)

**Figure 5:**
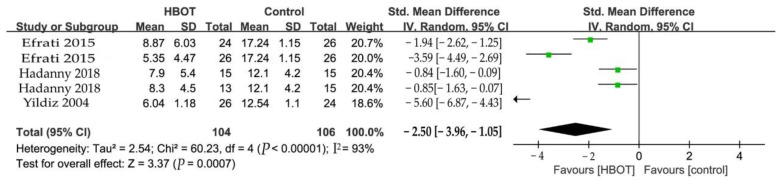
Forest plot for the comparison of TPC at the end of treatment, showing the effect favoring hyperbaric oxygen therapy [12,19,24].

TPC was measured by two studies as the outcome at the end of HBOT sessions (Figure 5). And two studies were assigned to treated and crossover groups. Compared with the control group, HBOT improved the clinical efficacy of TPC by −250% (SMD = −2.50, 95% CI −3.96 to −1.05, *p* = 0.0007). There was considerable heterogeneity between these studies (I^2^ = 93%), and a random-effects model was used. 

#### 3.5.4. Side Effects

**Figure 6:**
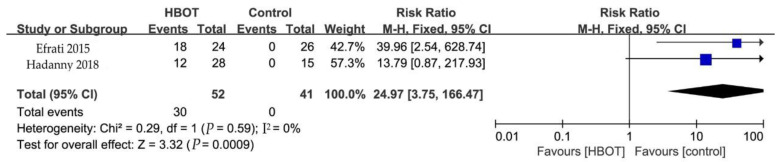
Forest plot for the side effects, showing the effect favoring hyperbaric oxygen therapy [19,24].

Two studies described side effects at the end of HBOT sessions (Figure 6). The most side effects were middle ear barotrauma. HBOT significantly increased the incidence of side effects (RR = 24.97, 95% CI 3.75 to 166.47, *P* = 0.0009) with no heterogeneity (*P* = 0.59, I^2^ = 0%). A fixed-effects model was adopted.

## 4. Discussion

Despite widespread application, there are very few RCTs assessing HBOT benefits for patients with FMS. This is the first systematic review and meta-analysis of RCTs on HBOT for FMS. This meta-analysis presents a novel line of evidence that HBOT can benefit FMS with FIQ and TPC at the end of treatment. However, the findings of the current meta-analysis indicate that HBOT therapy did not improve the pain scores. The use of HBOT, compared to placebo, was associated with a significant increase in side effects. Nevertheless, patients with mild barotraumas as side effects resolved spontaneously and completed the treatment protocol.

There are a number of studies supporting the use of HBOT to decrease inflammation and pain in rat models [27,28,29,30,31,32]. However, level 1 evidence for HBOT favors pain relief and inflammation decreases is lacking. RCTs with large sample sizes have not been conducted. In 2004, Yildiz et al. [12] conducted a randomized controlled study with 50 patients to evaluate the effect of HBOT on fibromyalgia, and the results demonstrated a decrease in pain scores. In addition, 49 women with FMS took part in Izquierdo-Alventosa’s [25] randomized controlled trial; the result showed that the perceived pain significantly improved only in the HBOT group as opposed to the physical exercise group and control group. However, there are no similar results from the meta-analysis after combining the data. The reason may be related to the use of different VAS scoring standards in the two included studies. Furthermore, as a result of the subjects’ inadequate physical exercise routines, they were unable to adjust to the pace of the recommended physical activity. This lack of adaptation could potentially result in excessive exertion and subsequently cause persistent sensations of discomfort and pain. 

Repeated treatment with HBOT produced a two-stage antinociceptive response in rat models [33]. After the last HBOT treatment, mice treated with HBOT for 60 min at 3.5 ATA four times a day had a robust antinociceptive response (suppression of abdominal constrictions) for up to 6 h. When the rats underwent abdominal contraction testing after additional time intervals, the antinociceptive effect of HBOT was not obvious for 12 h, and it began to re-emerge 24 h later after the last treatment of HBOT and lasted for up to 14 days post-treatment. This may also be the reason for the inconsistencies in pain relief among the RCTs included in different sessions. 

Three studies [12,19,24] described changes in tenderness thresholds, all showing significant increases in pain thresholds after HBOT treatment. However, the tenderness thresholds of the three studies were obtained from different parts of the body, so we did not perform a pooled analysis. The two studies [19,24] that utilized the FIQ (Fibromyalgia Impact Questionnaire) included patient assessments for various symptoms, such as difficulty with work, pain, fatigue, morning fatigue, stiffness, anxiety, and depression. At the conclusion of treatment, all patients experienced a significant improvement in their physical function. This seems to be another confirmation that HBOT can relieve pain in patients with FMS.

The etiology of FMS is still unknown, with several hypotheses proposed. It has been hypothesized that local hypoxia may lead to degenerative muscle changes that lead to chronic pain [34], which in turn leads to decreased ATP and elevated lactate concentrations. HBOT improves muscle oxygenation in fibromyalgia patients by improving the body’s oxygen tension, thus restoring aerobic metabolism and correcting local tissue hypoxia and acidosis [12]. Furthermore, HBOT causes hyperoxia, amplifying the diffusion gradient of oxygen between tissue and cells, thus raising plasma dissolved oxygen to levels beyond the physiological requirements of many tissues at rest [35]. A recent study examined 185 FMS patients with sicca and/or xerostomia symptoms and found a link between auto-antibodies and FMS, with one-third of them positive for a biomarker of Sjögren’s syndrome, and most of them were also positive for one or more tissue-specific auto-antibodies [36]. Guggino et al. [37] confirmed the involvement of the immune system in the pathogenesis of FM and highlighted the impact of HBOT treatment, especially the changes in pro-inflammatory cytokines produced by the CD4 T cell subpopulation. These may also be the reasons why HBOT can improve symptoms in patients with FMS. Furthermore, according to the study by Efrati et al. [19], HBOT can correct abnormal brain activity associated with pain. This involves reducing activity in overactive areas, primarily located in the back of the brain, and increasing activity in inactive areas, mainly located in the frontal lobe. These findings align with current understanding of how the brain responds to pain.

High heterogeneity (0% to 96%) was noted between included studies due to several factors. Firstly, the sustained effectiveness of HBOT is closely related to the time and session of treatment. Notably, exposure times and press for HBOT varied. Secondly, the definition of “quality of life” was not consistent due to different economic levels and regional cultures. Thirdly, due to the special hallmarks of the intervention, almost all included randomized controlled studies were not perfect in terms of blind methodology. Finally, the proportion of females in all the studies was higher. Men and women may have different levels of pain threshold. These factors may be contributive to the inter-group differences, affecting the entire heterogeneity of included studies. 

The limitations of this systematic review are mainly ascribed to the quantity and quality of included studies. This meta-analysis, including four RCTs, could not identify the underlying factors leading to heterogeneity. Therefore, it is necessary to conduct large-sample RCTs and additional studies for subgroup analyses in the future. 

## 5. Conclusions

The systematic review and meta-analysis support the effectiveness of HBOT in TPC and the improvement of functions in FMS with emerging evidence from 163 participants. Although HBOT has some side effects, it does not cause serious adverse consequences. Prolonged treatment sessions may help with pain relief. However, significant differences in the quality and heterogeneity of currently available evidence should be noted. Larger sample RCTs with unified regimens and longer follow-up times are needed.

## Figures and Tables

**Figure 2:**
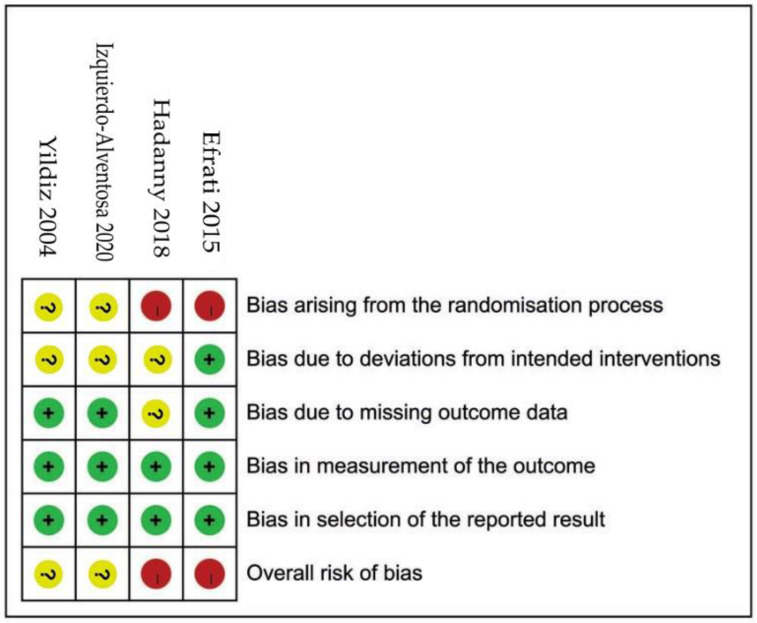
ROBs (2.0) within the included studies. Green circle and ‘+’, low risk; red circle and ‘−’, high risk; yellow circle and ‘?’, unclear risk [12,19,24,25].

**Table 1:** Search strategy and results.

Database	Step	Search Algorithm	Items
**PubMed**	#1	“Fibromyalgia” [Mesh Terms]	8800
	#2	“Musculoskeletal Disease” [ Mesh Terms]	1849
	#3	“Chronic Pain Syndrome” [All Fields]	705
	#4	“FMS“ [All Fields]	9726
	#5	(((#1) OR (#2) OR (#3) OR #4)	1,139,600
	#6	“oxygen therapies, hyperbaric” [ Mesh Terms]	12,098
	#7	“Hyperbaric Oxygenation” [All Fields]	12,467
	#8	“Hyperbaric Oxygenations” [All Fields]	12,468
	#9	“Hyperbaric Oxygen Therapies” [All Fields]	12,661
	#10	“Hyperbaric Oxygen Therapy” [All Fields]	14,330
	#11	“HBOT” [All Fields]	907
	#12	(((((#6) OR #7) OR #8) OR #9)OR #10) OR #11)	619
	#13	#5 AND #12	1137
**Embase**	#1	‘fibromyalgia’/exp	21,930
	#2	‘musculoskeletal disease’	38,748
	#3	‘chronic pain syndrome’	1223
	#4	‘FMS’	13,022
	#5	#1 OR #2 OR #3 OR #4	72,195
	#6	‘Hyperbaric Oxygen Therapy’	19,726
	#7	‘HBOT’	1220
	#8	#6 OR #7	19,743
	#9	#5 AND #8	66
**Cochrane**	#1	MeSH descriptor: “Fibromyalgia” explode all trees	1429
	#2	MeSH descriptor: “Musculoskeletal Diseases” explode all trees	42,086
	#3	Chronic Pain Syndrome: ti, ab, kw (Word variations have been searched)	303
	#4	FMS: ti, ab, kw (Word variations have been searched)	704
	#5	#1 or #2 or #3 or #4	45,416
	#6	MeSH descriptor: [Balneotherapy] explode all trees	261
	#7	Spa therapy:ti,ab,kw (Word variations have been searched)	122
	#8	Thermal water:ti,ab,kw (Word variations have been searched)	79
	#9	Balneology:ti,ab,kw (Word variations have been searched)	211
	#10	BT:ti,ab,kw (Word variations have been searched)	1373
	#11	#6 or #7 or #8 or #9 or #10	1848
	#12	(#5 and #11) restricted as clinical trials	52
**Ovid Medline**	#1	Fibromyalgia. mp.	10,642
	#2	Musculoskeletal disease. mp.	896
	#3	Chronic Pain/	16,300
	#4	#1 or #2 or #3	27,075
	#5	oxygen therapies, hyperbaric. mp.	0
	#6	Hyperbaric Oxygenation/	12,083
	#7	Hyperbaric Oxygen Therapy. mp.	3382
	#8	#5 or #6 or #7	12,614
	#9	#4 and #8	19
**Ovid PsycINFO**	#1	Fibromyalgia. mp.	3596
	#2	exp Musculoskeletal Disorders/	18,206
	#3	exp Chronic Pain/	13,747
	#4	#1 or #2 or #3	31,384
	#5	Hyperbaric Oxygen Therapy. mp.	122
	#6	exp Oxygenation/	962
	#7	#5 or #6	1082
	#8	#4 and #7	5
**Clinicaltrials.gov**	#1	Fibromyalgia	1088
	#2	Musculoskeletal Diseases	18,733
	#3	Diseases	273,674
	#4	Musculoskeletal	19,065
	#5	Chronic Pain Syndrome	45
	#6	Pain Syndrome	1612
	#7	Chronic Pain	2003
	#8	Syndrome	28,720
	#9	Chronic	25,425
	#10	Pain	21,325
	#11	FMS	1167
	#12	Hyperbaric Oxygenation	171
	#13	Oxygenation	1136
	#14	Hyperbaric	410
	#15	Hyperbaric Oxygen Therapy	171
	#16	Oxygen Therapy	3596
	#17	Hyperbaric Oxygen	208
	#18	Therapy	140,000
	#19	Oxygen	3833
	#20	HBOT	91
	#21	Musculoskeletal Diseases OR Fibromyalgia OR Chronic Pain Syndrome OR FMS|Hyperbaric Oxygenation OR Hyperbaric Oxygen Therapy OR HBOT	16

**Table 2:** Detailed information and characteristics of included studies.

Studies	Country	Mean Age (Y)		Female/ Male		Sample Size (n)		Duration (Y)		Intervention		Outcomes	Follow-Up
		HBOT	con	HBOT	con	HBOT	con	HBOT	con	HBOT	con		
Efrati 2015 [19]	Israel	50.4 ± 10.9	48.1 ± 11.1	NA	NA	24	26	6.75 ± 5.9	6.2 ± 5.1	hyperbaric oxygen therapy at 2.0 ATA. 40 daily sessions, 5 days/week, 90 min each.	no treatment	TPC (of 18),Pain threshold, FIQ, SF-36	2M
Hadanny 2018 [24]	Israel	48.3 ± 10.6	43.1 ± 10.6	30	0	15	15	NA	NA	hyperbaric oxygen therapy at 2.0 ATA and psychological therapy. 60 daily sessions, 5 days a week. 90 min each.	psychological therapy	WPI, TPC (of 18), FIQ, SF-36	3M
Yildiz 2004 [12]	Turkey	40.46 ± 4.79	39.88 ± 4.71	35	15	26	24	4 ± 1.1		hyperbaric oxygen therapy at 2.4 ATA. 15 sessions, one session per day for 5 days of the week, 90 min each.	breathed air at 1 ATA for 90 min	TPC, 6MWT, VAS, Pressure pain threshold	3W
Izquierdo-Alventosa 2020 [25]	Spain	NA	NA	33	0	17	16	NA	NA	hyperbaric oxygen therapy at 1.45 ATA. 40 sessions, five sessions per week, 90 min each.	usual medication	VAS, pressure pain threshold	10W

HBOT = Hyperbaric Oxygen Therapy, con = control, NA = not available, Y = years, M = months, W = weeks, ATA = atmosphere absolute, WPI = Widespread pain index, FIQ = Fibromyalgia functional impairment, 6MWT = Fatigue following the completion of the 6-min walking test, VAS = visual analog scale.

## Data Availability

The datasets supporting this systematic review and meta-analysis are from previously reported studies and datasets which have been cited. The processed data are shown in Table 2 and Figure 3 to Figure 6.
